# Striking a new balance: A qualitative study of how family life has been affected by COVID-19 *

**DOI:** 10.1590/1518-8345.6705.4044

**Published:** 2023-11-03

**Authors:** Mayckel da Silva Barreto, Francielle Renata Danielli Martins Marques, Adriana Martins Gallo, Cristina Garcia-Vivar, Lígia Carreira, Maria Aparecida Salci

**Affiliations:** 1 Universidade Estadual de Maringá, Departamento de Enfermagem, Maringá, PR, Brasil.; 2 Bolsista da Coordenação de Aperfeiçoamento de Pessoal de Nível Superior (CAPES), Brasil; 3 Universidad Pública de Navarra, Departamento de Ciencias de la Salud, Pamplona, NA, España; 4 Bolsista do Conselho Nacional de Desenvolvimento Científico e Tecnológico (CNPq), Brasil

**Keywords:** Pandemics, COVID-19, Family, Family Relations, Family Nursing, Qualitative Research, Pandemias, COVID-19, Familia, Relaciones Familiares, Enfermería de la Familia, Investigación Cualitativa, Pandemias, COVID-19, Família, Relações Familiares, Enfermagem Familiar, Pesquisa Qualitativa

## Abstract

**Objective:**

to examine the repercussions of the pandemic on the family system by focusing on the perspective of family members who contracted and experienced COVID-19.

**Method:**

an exploratory study with a qualitative approach conducted with 27 individuals who had COVID-19. Data collection took place through telephone interviews that were audio-recorded and guided by a semi-structured instrument. Data analysis was based on an inductive process supported by Reflexive Thematic Analysis.

**Results:**

the pandemic and illness of a family member acted as driving forces generating new and intense movements in the family system. Initially, they noticed negative repercussions such as concerns, fear, anguish, stress, distancing and social isolation. As time progressed and by using technologies to ease communication, they began to perceive positive repercussions such as increased proximity, strengthening of ties, development of new roles and care. The families also identified recovery of a balanced position, with return of certain readjustment in family dynamics and functioning.

**Conclusion:**

health professionals need to recognize that the COVID-19 disease has imposed repercussions on family systems, proposing interventions that help families face this moment and more easily recover a balanced position for their functioning.

## Abstract (es)

**Objetivo:**

examinar las repercusiones de la pandemia en el sistema familiar enfocándose en la perspectiva de familiares que han contraído y padecido COVID-19.

**Método:**

estudio exploratorio de enfoque cualitativo realizado con 27 personas que tuvieron COVID-19. Los datos se recolectaron por medio de entrevistas telefónicas que se grabaron en audio y estuvieron guiadas por un instrumento semiestructurado. El análisis se basó en un proceso inductivo respaldado por Análisis Temático Reflexivo.

**Resultados:**

la pandemia y el hecho de que un familiar contrajera la enfermedad fueron fuerzas impulsoras que generaron movilizaciones nuevas e intensas en el sistema familiar. Inicialmente, notaron repercusiones negativas como preocupaciones, temor, angustia, estrés, distanciamiento y aislamiento social. Con el paso del tiempo y empleando tecnologías para facilitar la comunicación, comenzaron a percibir repercusiones positivas como más cercanía, fortalecimiento de vínculos, desarrollo de nuevos roles y cuidado mutuo. Las familias también identificaron que recuperaban una posición de equilibrio, con retorno de cierto reajuste en la dinámica y el funcionamiento familiar.

**Conclusión:**

los profesionales de la salud deben admitir que la enfermedad por COVID-19 ha generado repercusiones en los sistemas familiares, además de proponer intervenciones que ayuden a las familias a hacer frente a este momento y a recuperar más fácilmente una posición de equilibrio para su buen funcionamiento.

## Abstract (pt)

**Objetivo:**

examinar as repercussões da pandemia no sistema familiar a partir da perspectiva dos familiares que contraíram e vivenciaram a COVID-19.

**Método:**

estudo exploratório com abordagem qualitativa realizado com 27 indivíduos que tiveram COVID-19. A coleta de dados ocorreu por meio de entrevistas telefônicas gravadas em áudio e guiadas por um instrumento semiestruturado. A análise dos dados baseou-se num processo indutivo apoiado na Análise Temática Reflexiva.

**Resultados:**

a pandemia e o adoecimento de um familiar atuaram como motores geradores de novos e intensos movimentos no sistema familiar. Inicialmente, perceberam repercussões negativas como preocupações, medo, angústia, estresse, distanciamento e isolamento social. Com o passar do tempo e com o uso das tecnologias para facilitar a comunicação, os familiares começaram a perceber repercussões positivas como maior proximidade, fortalecimento dos laços, desenvolvimento de novos papéis e cuidado. As famílias também identificaram recuperação de uma posição equilibrada, com retorno de certo reajuste na dinâmica e funcionamento familiar.

**Conclusão:**

os profissionais de saúde precisam reconhecer que a doença COVID-19 impôs repercussões nos sistemas familiares, propondo intervenções que ajudem as famílias a enfrentar esse momento e recuperar mais facilmente uma posição equilibrada para seu funcionamento.

**Highlights:**

**(1)** The family system was affected in terms of its dynamics and functioning during the pandemic

**(2)** Initially, families moved towards negative repercussions

**(3)** The more severe the COVID-19 condition, the more negative repercussions noticed by the families.

**(4)** There was also a movement towards positive repercussions.

**(5)** Families began to identify recovery of a balanced position

## Introduction

In December 2019, a new viral respiratory disease emerged in China, which spread rapidly and alarmingly across the planet and was called COVID-19 by the World Health Organization ^(^ 1 ^)^. Some of the strategies most widely employed by different countries aimed at reducing the spread of the virus included mandatory quarantine of suspected and confirmed cases, social distancing and establishment of lockdowns to avoid crowding and viral circulation ^(^ 2 ^)^. Thus, the pandemic affected different human life spheres and led people to remain in their homes, unable to perform their usual activities such as going to school, working and participating in social, cultural and sports meetings ^(^ 1 ^)^. 

As a problem of global proportions, the COVID-19 pandemic has caused social, economic and political impacts on various societies and, resonantly, on families, causing stress, intensifying vulnerabilities and changing the way in which its members related to each other ^(^ 3 ^-^ 4 ^)^. In addition, other tangible losses affecting families include income, access to means and resources, and planned activities or celebrations ^(^ 5 ^)^. In turn, family relationships needed to adapt to a new reality permeated by doubts, insecurities, new junctions and different ways of organizing habits and routines, changing customs and pre-established family relationships ^(^ 3 ^)^. 

Recent studies ^(^ 2 ^,^ 6 ^-^ 7 ^)^ showed that the pandemic and the measures adopted to cope with it affected people’s mental health and led to the emergence of symptoms such as stress, anxiety and depression. In addition, when there is viral infection within the family, the barriers to preserving mental health end up being even greater, as this has varied consequences, whether physical, financial or emotional ^(^ 8 ^)^. Moreover, COVID-19 sometimes triggered sequential grief in the same family, causing confrontations and difficulties for them to adapt to successive and abrupt losses of loved ones and coexistence ^(^ 9 ^)^. This scenario has demanded from families a structural and developmental reorganization process in the search for protecting ties and functioning of the family system ^(^ 10 ^)^. 

Currently, the situation revolves around identifying and minimizing the consequences imposed by the pandemic on life in society. This is because the disease has shown that its implications go beyond aspects related to health or economics, as it reverberated in the field of relationships and affections, by directly modifying the way in which people relate to each other and live as social beings and families ^(^ 11 ^)^. On the other hand, more studies are required to show how people affected by COVID-19 noticed the changes in family relationships due to the pandemic and disease. This will allow us to understand, in a more fruitful way, how such repercussions influenced functionality of family systems in crisis and how these repercussions can be considered by health professionals for the development of interventions that unite the family system. Thus, this study aimed at examining the repercussions of the pandemic on family systems by focusing on the perspective of family members who contracted and experienced COVID-19. 

For this study, the theoretical framework used was the theory of Family Systems Nursing (FSN) ^(^ 12 ^)^. The family is understood as a care system and unit, comprised by several subsystems and, at the same time, comprising a larger suprasystem, that is, the family unit is greater than the sum of its parts (its individual members). Circularity and reciprocity influence the interrelation and interdependence among its members. The system is immersed in specific cultural and social contexts that guide meanings and life experiences. When a family member is affected (for example, in this case by the COVID-19 diagnosis), so are the others, albeit in different ways, as they might experience fear of loss or sequelae from the COVID-19 disease ^(^ 12 ^)^. In this regard, the FSN science and practice are based on the premise that the health-disease process affects the family system – including its structure, development and function – and influences the health and well-being of each family member. With family dynamics and functioning modified, its members tend to seek a balance between change and stability ^(^ 12 ^)^. 

Furthermore, it is crucial to acknowledge the significance of circularity within the family system. Circular relationships denote bilateral connections between system elements, characterized by interactive sequences that present circular patterns. Additionally, the concept of feedback plays a pivotal role in maintaining circular functioning, as it eases circulation of information among system components. Negative feedback mechanisms work to uphold homeostasis, whereas positive feedback mechanisms respond to systemic changes ^(^ 12 ^)^. By considering the principles of circularity and feedback, it becomes evident that the family unit dynamics and functionality exert an ongoing influence on evolution of the COVID-19 health-disease process and its broader ramifications. This balance is inherently dynamic, as the family undergoes transformative changes as a result of experiencing the disease ^(^ 12 ^)^. Therefore, it is crucial to recognize that the family no longer remains unchanged after facing COVID-19 but that it rather undergoes a significant transformation ^(^ 12 ^)^. 

When employing this theoretical framework, studies on and with families are able to produce knowledge about individual and family functioning through the analysis of family interactions and functioning and how the available internal and external family resources are used to achieve homeostasis ^(^ 12 ^)^. Family homeostasis involves regulation and adjustment of various dynamics, roles, rules and communication patterns within the family unit to preserve its balance and functioning ^(^ 12 ^)^. This phenomenon is not static but it involves a constant back-and-forth movement between change and stability ^(^ 12 ^)^. Therefore, in view of the recognition and wide application of this framework in the Nursing practice and in research, this study adopts the premises of the FSN theory to explore the repercussions of COVID-19 on the family system. 

## Method

### Type of study

This study is part of a multicenter sequential explanatory and mixed-methods project that received federal funding ^(^ 13 ^)^ and was entitled “Blinded for review” with the aim of exploring and analyzing predictors and consequences of COVID-19 in adults and aged people who developed mild, moderate or severe forms of the disease. For this study clipping, results regarding the qualitative and exploratory approach were presented. The data are allusive to the family, but exclusively from the perspective of patients who had COVID-19. The patients’ families were not interviewed. The Consolidated Criteria for Reporting Qualitative Research (COREQ) checklist was used to report this qualitative study ^(^ 14 ^)^. 

### Study locus and participants

The locus was the state of Paraná, located in the Brazilian South region, with an estimated population of 11.6 million inhabitants distributed in 399 municipalities and four health macro-regions. The Human Development Index is 0.749 – ranking 5 ^th^ in the national ranking of states ^(^ 15 ^)^. 

The study participants were recruited to comprise a convenience sample, of wide variation, from the baseline of the COVID-19 Cohort. The researchers possess an 18-month follow-up database of patients who experienced COVID-19 during 2020, extending beyond the acute phase of the disease. To compile data for these patients, the researchers initially accessed official reporting databases. Specifically, mild cases, which were treated in outpatient clinics, were obtained through the *Notifica COVID Paraná* registry. On the other hand, moderate and severe cases, involving patients admitted to a ward or ICU, were extracted from the Influenza Epidemiological Surveillance Information System ( *Sistema de Informação de Vigilância Epidemiológica*, SIVEP-Influenza) ^(^ 13 ^)^. 

To address the need for wide variation in characteristics of the participants in this qualitative study, the following inclusion criteria were applied: being over the age of 18; having had COVID-19 between March and December 2020; having been classified with COVID-19 in mild or moderate/severe forms during the hospitalization period; and having sufficient communication ability. As exclusion criteria, we considered people who did not have access to the Internet and/or to electronic devices that allowed conducting the interviews. Twenty-seven patients were invited to participate in the study and there were no refusals or withdrawals.

The classification of the mild form consisted of people who were treated due to COVID-19 on an outpatient basis, for presenting mild signs or mild respiratory tract infection; in turn, the moderate form corresponded to those who required hospitalization in ward units, with the need for non-invasive ventilator support; and the severe form was represented for those in need of Intensive Care Unit (ICU) for hemodynamic stability and invasive mechanical ventilation ^(^ 16 ^-^ 17 ^)^. 

### Data collection

Data collection took place between March and May 2022. The interviews were conducted through recorded telephone calls. This is because the participants were from several cities in the state of Paraná selected from a mixed-methods cohort study ^(^ 13 ^)^. The interviews were guided by a semi-structured instrument, previously validated by ten specialists with recognized teaching and research profiles in the health area. The first 10 interviews were pilot-tested and were also included in the study. The guiding question was as follows: “ *Please tell me about the main changes in your family life and family relationships/ties during the COVID-19 pandemic*”, which was supplemented by support questions. 

A single interview was conducted with each participant. The interviews lasted a mean of 15 minutes and were conducted by two researchers, a PhD in Nursing and another PhD student in Nursing with extensive experience in qualitative research, without prior attachment to the participants, except that they are already part of the follow-up cohort. Initially, the interviewers introduced themselves and reiterated the importance and reasons for the interviewees to continue participating in the research, as they had already formalized their participation in the COVID-19 Cohort at a previous moment.

All interviews were transcribed in full. Throughout the data collection period, field notes were also recorded according to the researchers’ perceptions regarding quality of the interviews, aspects related to the participants’ tone of voice and nonverbal communication, and the need to add new questions to expand collection of the information. Data collection and analysis were simultaneous until reaching data saturation.

### Data analysis

The interviews were analyzed according to the six stages of Reflexive Thematic Analysis ^(^ 18 ^)^. In the first stage, superficial and in-depth readings of the transcribed interviews were performed. In the second stage, the initial codes were identified inductively from the interviews and 234 codes emerged from the initial analysis. In the third stage, the similarities and differences between the initial codes were identified. The analysis of the initial codes was developed considering the similarities and differences between the participants’ reports. In the fourth stage, the themes were improved according to the FNS conceptual framework ^(^ 12 ^)^. In the fifth stage, the themes were named and refined. Finally, in the sixth stage, the Results section was developed. 

The first and second stages were developed by three researchers who were involved in data collection; and the subsequent stages were discussed and validated by the entire research team, another three researchers experienced in qualitative data analysis and who met regularly. The MAXQDA software was used as an aid in the coding process. Field notes related to the interview context supported data analysis elaboration. The analytical process was triangulated through the use of field notes linked to the transcription of the interviews. These interviews were witnessed by three different research group members, ensuring consistency within the same methodological framework. At the end of data analysis, the researchers asked the participants to validate their testimonies, and all agreed with the results. Recruitment of the participants was interrupted when, after discussion and consensus among the researchers, data saturation was reached ^(^ 19 ^)^. 

### Methodological rigor

In order to imprint methodological rigor, data collection, analysis and interpretation were independently conducted by three researchers, guided by reflexivity, where previous assumptions were recognized and left in suspension ^(^ 20 ^)^. During the analysis, in case of inconsistencies, the team of researchers met and discussed the analytical and interpretive process of the data, reaching consensus. Transferability and confirmability were ensured by detailed explanations about the research locus and participants. Finally, credibility and reliability were ensured by maintaining an audit trail, which guaranteed that all research documentation was accessible for future consultations ^(^ 21 ^)^. 

### Ethical aspects

The study was approved by the Ethics Committee of the signatory institution. Once the participants were recruited from the baseline of the COVID-19 Cohort, all had already consented to participate in the research and were already in possession of the Informed Consent Form. To respect the participants’ anonymity, the testimonies were identified by code names with the letter “I” for Interviewee, followed by a number referring to the order in which they were included in the research (for example, I01).

## Results

The study included 27 individuals diagnosed with COVID-19, 17 of them male. Their age ranged from 33 to 80 years old, although most of them were between 51 and 70 years old (18 cases). Most were white-skinned (23), married (15), had more than eight years of study (19), and were Catholics (20). As for COVID-19, most of the individuals were classified with severe disease and required hospitalization in the ICU (19). Four individuals required hospitalization in the ward and another four needed outpatient care.

**Figure 1 -:**
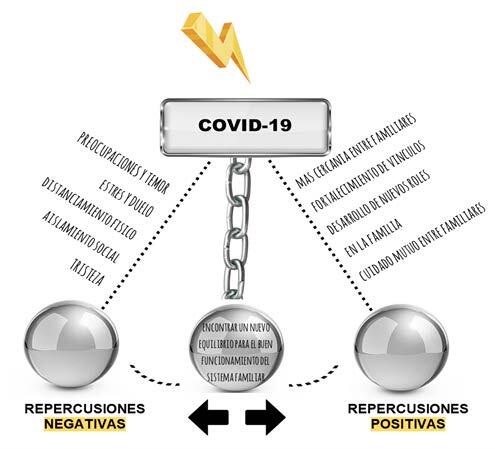
Diagram representation of the themes identified in the study

Three themes were identified from data analysis, namely: “Negative repercussions of the COVID-19 pandemic on the family system”; “Positive repercussions of the COVID-19 pandemic on the family system”; and “Striking a new balance for functioning of the family system”, which reveal that the family system in this study is represented by a pendulum ( Figure 1). When reflecting on the repercussions of the COVID-19 pandemic, the families draw a comparative parallel with the period before its occurrence and show that, prior to the pandemic, they followed their usual dynamics and functioning. 

The pandemic situation, the illness of a family member and the social, cultural, educational, economic and political repercussions of the pandemic, to some extent shared by society as a whole, were driving forces generating a new and intense movement in the family system. The families initially moved towards the extreme that represents the negative repercussions, perceiving concerns, fear, anguish and stress. In addition, they experienced physical distancing from the extended family and social isolation, which triggered sadness. The more severe the COVID-19 disease, the more negative repercussions noticed by the family, especially due to the hospitalization time and the need for intensive care and greater adaptations to favor recovery in the post-COVID-19 period. As time progressed and by using information technologies as a strategy to promote communication, family systems also began to perceive positive repercussions from the pandemic and the disease, such as increased proximity, strengthening of ties, development of new roles and care among its members.

Faced with changes in the pandemic epidemiological pattern and learning to socialize with its repercussions, the families began to identify recovery of balanced position, with the return of homeostasis to family life, its dynamics and functioning. The swinging view does not depict a perspective of polarized families. Instead, it illustrates the oscillation along a continuum, reflecting the process of experiencing the COVID-19 repercussions within the family system. This oscillation traverses from one end to the other, capturing the varying impacts and effects caused by the disease.

### Negative repercussions of the COVID-19 pandemic on the family system

The COVID-19 pandemic imposed a series of negative repercussions on families. The concern with the health of family members and the fear of contracting the virus or even transmitting it show the globality of the family and were responsible for triggering new habits and behaviors (morphogenesis), such as social isolation and distancing from family members, friends and neighbors. Sadness was a feeling commonly identified in families when experiencing negative repercussions. *We got more distanced (the family relationship), for example, my sisters who came here, relatives who came to visit more here at home, they all disappeared (I21). I miss family life. I have a very sick sister too that I can’t go visit anymore, so it’s very difficult to stay away from our family, it causes me much sadness (I19)*. 

The pandemic and its consequences were responsible for changing the globality, feedback and circular relationships between members of the family system, as personal stress affected communication and relationships between its members. *With the pandemic we became more nervous here at home, anything anyone says the other is already rude, at the beginning of the pandemic, the relationships were very difficult (I22). After COVID-19 I ended up separating from my wife, we began to live more time in the home and kept quarreling, in conflict, it was very complicated. Now I want peace (I01)*. 

Many families signaled financial changes, even if not so sharp for some; there were changes in people’s purchasing power, triggering insecurity in relation to the future due to the financial instability of the moment. *In financial matters, I don’t earn half the money I earned before. I lost my job and it’s difficult to get another one. In addition, everything is very expensive, I can’t buy the things I used to buy. It’s hard (I02). That’s all I’m finding bad: not to be working! Everything else is fine, our family is organizing, no need to rush. But the doctor gave me four or five expensive drugs. I bought them about three, four times and then didn’t take them and I’m not taking them anymore (I14)*. 

The negative repercussions were more intensely perceived in families where the person affected by COVID-19 required more hospitalization time and intensive care, as well as among those that demanded greater care for recovery after prolonged COVID-19. This was especially due to the family fear of losing their loved one during hospitalization, namely in the Intensive Care Unit, and to the possibility of permanent sequelae. *At first it was quite difficult for everyone, they were afraid of death when I was in the ICU, afraid of my lungs not working well again, of needing to live on oxygen. Then when I was discharged from the hospital, I needed a lot of help. At that moment I still wasn’t able to walk. So I had to come to my sister’s house to get physical therapy. I mobilized the whole family. She made available a space in her house so I could undergo all the recovery (I12). I think that the worst of all was having gone to the ICU, it was at that time that my family was most disrupted. Fear was significant and the doubts were many (I05)*. 

### Positive repercussions of the COVID-19 pandemic on the family system

The COVID-19 pandemic and illness of one of the family members were also responsible for triggering positive repercussions in families. To foster homeodynamics within the family system, it was imperative to ease care among the family members, strengthen interpersonal ties, enhance proximity and cultivate the development of new roles. These measures were crucial in promoting dynamic balance and adaptability within the family unit, enabling it to effectively navigate the challenges posed by external factors such as COVID-19. *We’ve always been very close, but with the pandemic we’re even closer. My son lives a little far from my house and he came to meet me every morning to give me a bath and my daughter started to come too, as I need to wear a diaper, she started changing my diaper every night. They did the son/daughter part right, my husband even learned to cook. After I became ill with COVID-19, the concern that they (relatives) have with me increased (I04). I have three daughters and I get along with them, but I think that after the initial fear, the pandemic helped because they got even closer to us, they came to take care of me, to shop because my wife couldn’t go alone (I15)*. 

Death resulting from the COVID-19 pandemic revealed the nonsummativity of the family system. This is because its impact was felt throughout the system, in the subsystems (mother-children, husband-wife), as well as in everyone in a singular way. However, at the same time, it showed the importance of family globality and cohesion so that it can continue to function, based on the help and mutual support between its members. *Our life changed a lot with the pandemic, because she (wife) died due to COVID-19 and then we were alone, just me and the children. They (children) live with me, I have a little child. We have to follow life, relearn to live. We’re sad, the children are sad, because they miss their mother. Not having my wife is also bad. But we have to overcome it, one helps the other, we support each other and so we keep going (I11)*. 

Throughout the process of morphogenesis and adaptations within the family system, the use of technologies played a prominent role, as it was understood as a strategic resource that favored maintenance of family relationships and ties, even during hospitalization due to COVID-19. *Technology helps a lot. Now it’s much better than before, no doubt. Technology has helped in every way, we can communicate much more easily with our family members, this reduces the distance (I18). When I was in the ICU, the social worker made a video call with my family and it helped me a lot when I was in the ICU (I07)*. 

It was important for families to preserve their ties and be able to communicate with their loved ones during hospitalization. It was a way of being close to them and promoting emotional closeness among their members by sharing their fears, as well as the joys of recovery.

### Striking a new balance for functioning of the family system

The participants felt the changes and negative repercussions in the family system, especially in the first months of the pandemic and among those with greater severity in their clinical condition resulting from the COVID-19 infection. However, they revealed that, little by little, the families began to learn how to deal with the pandemic situation and, thus, return to a homeodynamic balanced position for proper family functioning. *At first, we were more worried. When I was in the ICU the family was very apprehensive. But now the pandemic has calmed down, we’re calmer and learning every day to live with it (I05). The pandemic has changed our lives a lot, there were no birthday parties. Last year I lost my grandparents and an uncle. So the pandemic affected a lot, because we were always very close, we used to have New Year’s Eve party. But now with this pandemic this didn’t happen anymore. In fact, now that we’re gradually returning to relate, to have our lives back to a more normal pattern and this has made a difference (I26)*. 

Similarly to the negative repercussions perceived by the participants, among the positive ones it was observed that, over time, the family system sought to recover a new balanced position for its functioning and circular relationship between its members. *My relatives were very apprehensive when I caught COVID-19, apprehensive about the disease situation and they took good care of me. In fact, at that time it seems that they had greater concern for me, now everything is more normal (I02). When I was admitted to the ICU, my brothers got closer to me. They were already close, only more at that time. But now everything is back to normal, each one has their things tofollow (I20)*. 

The families sought to achieve dynamic balance for proper functioning and to adapt to the new normality. Severity of the disease seems to be an important marker of distress, adaptations and family reorganization of the functioning pattern. Nevertheless, in the same way that hospitalization in an ICU triggered negative repercussions, it also brought greater proximity between the members. Over time, both the negative and the positive repercussions were mitigated to restore balance within the family. Through this transformative process of dynamic balance, the family underwent changes and adaptations, assuming new positions and roles. As a result, the family system evolved and occupied different positions, embracing a new configuration that eased its continued growth and resilience.

## Discussion

This study contributes to expanding our understanding by revealing the diverse effects of the COVID-19 pandemic on the dynamics and functioning of family systems. The findings indicate that COVID-19 severity correlates with the magnitude of the negative repercussions experienced by families. However, as families actively addressed these challenges, they gradually identified a path towards restoration of a new balance in their functioning. This highlights the families’ resilience and adaptive capacity in navigating and recovering from the impacts of the pandemic. In fact, there were important changes in the family life routine during the COVID-19 pandemic at a scale probably not seen since World War II ^(^ 22 ^)^. 

The negative consequences identified were especially characterized by fear of contracting the virus or spreading it to other family members, feelings of sadness and loneliness resulting from social distancing, and physical and mental stress signs. A number of studies have shown that people undergoing social isolation reported some stress degree and unregulated sleep ^(^ 23 ^-^ 24 ^)^. Such factors may have contributed to family disagreements during the pandemic, with an increase in quarrels, intolerance and difficulty in communication and understanding. 

One of the most salient consequences of the pandemic was the sudden increase in time shared between members of a couple, a result of isolation and remote work measures ^(^ 25 ^)^. Confinement-related stress was associated with the perceived decrease in relationship satisfaction ^(^ 25 ^-^ 26 ^)^. Thus, the pandemic imposed family structure fragmentation through divorces, whether consolidated or in the transition process, as pre-existence of this vulnerability in families increased susceptibility to social ruptures in moments of crisis and to the sequelae of the pandemic ^(^ 22 ^)^. Marital conflicts and divorces stood out in this period of uncertainty by generating implications for communication and challenges related to the creation of new rules, establishing trust between peers, financial rearrangement and coparenting ^(^ 27 ^-^ 28 ^)^. 

In addition to the distancing of marital relations, economic difficulties were also signaled as negative changes resulting from the pandemic, contributing to feelings of sadness and uselessness. Economic losses during the pandemic, such as unemployment and the impossibility of working, as well as costs related to the treatment itself, were factors that generated mental distress in families ^(^ 29 ^)^. Especially in the case of Brazil, this mainly affected families that were already in worse situations of socioeconomic vulnerability, and the federal government financial support to the less privileged families took time to be implemented ^(^ 30 ^)^. 

To some extent, the financial vulnerability evidenced in this study is close to the scenario presented by a North American study that details the assistance funded by the United States government which, although widely available during the pandemic and benefited the unemployed and needy, was not enough to cushion the negative financial impact of the pandemic for those who were financially more vulnerable ^(^ 31 ^)^. Further studies are required to analyze these aspects since the long-term indebtedness contracted by families during the pandemic will oftentimes still extend into future periods and will certainly affect family dynamics and functioning. 

Even with negative impacts in different family settings, this study pointed out positive repercussions related to the pandemic moment, such as proximity between family members, strengthening of ties, development of new roles, establishing greater care among its members and increasing the use of technologies to approach distant relatives, in order to maintain the ties and family relationships during hospitalization or social isolation moments. The literature points out that the positive family ties between people who exchange meaningful words of affection and mutual support, through messages for example, motivate and strengthen them so that they can move forward ^(^ 32 ^)^. 

In the pandemic context, it is remarkable that the use of information technologies served as an important strategy to minimize the harms related to the social isolation, quarantine, hospitalization and farewell processes ^(^ 9 ^,^ 33 ^)^; especially when fear and distress were amplified, in cases of patients in ICU and who could not receive visits, or even among relatives who were physically separated from hospitalized patients and awaited news from medical teams ^(^ 34 ^)^. In this regard, the social isolation experiences reinforced the importance of ensuring that families have the necessary resources to maintain social connections, such as digital access, allowing proximity when physical distance is necessary. 

Qualitative data from a large longitudinal cohort in Australia that examined the impact of the COVID-19 pandemic on male parents and their children provided a comprehensive summary of the everyday family reality and identified positive changes related to the pandemic, including higher quality of time spent with children ^(^ 35 ^)^. In the Brazilian and Australian cultures, it is typical for fathers to spend relatively fewer hours caring for their children when compared to mothers, and this is an aspect that was modified during the pandemic; there were also changes in work development and in the institution of emergency remote education. 

Research focusing on the negative and positive repercussions on families in response to the pandemic can contribute to understanding the impact of everyday life and modern environments on mental health and well-being. For example, questions about how parents can improve the new balance between work, personal and family life, sustaining the positive changes achieved during the pandemic, are fruitful and necessary to maintain long-term perceived benefits. Moreover, it is important to consider families that have not experienced this positive change so that they can identify ways to spend more time with their relatives ^(^ 36 ^)^. 

This study further revealed that, as the epidemiological pattern of the pandemic shifted and they began to feel safer and more confident, the families started to reintegrate extended relatives into their living arrangements. This marked a significant shift towards regaining a sense of balance in family functioning. However, it is important to note that this new equilibrium point was not a return to the pre-pandemic state but, rather, certain reconfiguration influenced by the experiences and transformations that the families had undergone. Thus, the families actively adapted and evolved, forging a new balance that incorporated the changes brought about by the pandemic. Other challenging circumstances in family life, such as the diagnosis of a serious disease like cancer ^(^ 37 ^)^ or the sudden loss of a loved one ^(^ 38 ^)^, have also been found to initially trigger negative repercussions. However, through the strong ties between family members and the process of reorganizing their functioning patterns, a new balanced position can be achieved, leading to positive outcomes. 

Ties are configured as one of the strengths of families, by providing a positive and vital perspective for the construction and development of family resilience ^(^ 32 ^)^. Resilience involves the potential for recovery, repair and growth of families experiencing important challenges in life, where the essential elements are hope, optimism, initiative, the art of seeing what is possible and perseverance ^(^ 39 ^)^. Such elements are necessary to develop individual and family capacity to adapt to crises, such as the pandemic triggered by COVID-19 and its consequences on family systems and their relationships. 

Thus, if on the one hand, the process of contracting COVID-19 interrupted people’s natural life path ^(^ 29 ^)^, especially among those families that experienced the most serious forms of the disease leading them to moments of solitude and reflection, on the other hand, it allowed them to reassess their self-esteem and recover the time lost with the family after the treatment ^(^ 29 ^)^, expanding the closeness and support moments. Therefore, policies and practices aimed at providing socio-emotional support to families, including support measures for maintaining family balance, may be relevant to improve well-being and reinforce positive changes in family and personal life. Research on the short- and long-term effects of the pandemic on family needs and resources is suggested. 

Moreover, it is necessary that health professionals, especially nurses, can support and empower families in adverse situations. One of the strategies for this would be the Family Strengths-Oriented Therapeutic Conversation, which is the center of the Family Nursing practice ^(^ 40 ^)^. Nonetheless, it is important to understand that a therapeutic Family Nursing conversation is not *per se* a single intervention. On the contrary, it configures a series of Family Nursing interventions offered in the context of a conversation and relationship between nurses and families, for which former must employ their therapeutic and communicational skills ^(^ 40 ^)^. Therapeutic conversations have contributed great results for families experiencing diverse health-disease situations, such as those of children diagnosed with chronic diseases ^(^ 41 ^)^ and of adults with heart failure ^(^ 42 ^)^ or stroke ^(^ 43 ^)^, promoting more mutual understanding and greater proximity among relatives. 

Among the study limitations, it is pertinent to point out the limits imposed by the pandemic itself, reflecting that the interviews were conducted remotely. However, it is noteworthy that the use of communication technologies favored the participation of people from different locations, expanding understanding of the phenomenon under investigation. In addition, the data only reflect the perspective of people who had COVID-19, without collecting information from other family members. Therefore, it would be important that future studies seek to know the view of entire family.

Despite its limitations, this study has made significant contributions to the Health and Nursing fields. The findings shed light on the impact of COVID-19 at various severity levels on family systems. It is evident that families navigate a delicate balance between negative and positive repercussions. Through circular interactions among family members, a new balance in family dynamics and functioning can be achieved. These results underscore the importance for healthcare professionals to collaborate with families in identifying the most effective interventions to ease adaptation to COVID-19 within their family unit. By considering each family’s unique needs and dynamics, health professionals can provide tailored support and empower families to cope with the challenges imposed by the disease.

## Conclusion

The results showed that, according to people who had COVID-19, the family system was moved in terms of its dynamics and functioning because of the pandemic and illness of a family member. Initially, they moved towards negative repercussions (concerns, fear, anguish, stress, physical distancing, social isolation and sadness). The more severe the COVID-19 condition, the more negative repercussions noticed by the family. Afterwards, there was also movement towards positive repercussions (increased proximity, strengthening of ties, development of new roles and care among its members). Faced with changes in the epidemiological pattern of the pandemic and learning to live with the virus and its repercussions, the families began to identify recovery of a balanced position, with the return of homeodynamics in family life, dynamics and family functioning.

In future pandemics, it will be crucial to acknowledge and understand the range of both negative and positive repercussions experienced by family systems. Consequently, it becomes essential to identify circularity points within family interactions and to collaborate with families in developing hypotheses and interventions that foster reorganization and establish a new equilibrium in family dynamics and functioning. Interventions aimed at promoting families’ well-being and healthy functioning must be prioritized, as they directly contribute to the overall health and resilience of all family members.
